# Trust and Mobility-Based Protocol for Secure Routing in Internet of Things

**DOI:** 10.3390/s22166215

**Published:** 2022-08-18

**Authors:** Syeda Mariam Muzammal, Raja Kumar Murugesan, NZ Jhanjhi, M. Shamim Hossain, Abdulsalam Yassine

**Affiliations:** 1 Department of Computer Science, Capital University of Science and Technology, Islamabad 44000, Pakistan; 2 School of Computer Science, SCE, Taylor’s University Lakeside Campus, Subang Jaya 47500, Malaysia; 3 Department of Software Engineering, College of Computer and Information Sciences, King Saud University, Riyadh 11543, Saudi Arabia; 4 Department of Software Engineering, Faculty of Engineering, Lakehead University, 955 Oliver Rd, Thunder Bay, ON P7B 5E1, Canada

**Keywords:** internet of things, IoT security, routing, RPL, RPL attacks, Rank, Blackhole, trust

## Abstract

In the Internet of Things (IoT), the de facto Routing Protocol for Low Power and Lossy Networks (RPL) is susceptible to several disruptive attacks based on its functionalities and features. Among various RPL security solutions, a trust-based security is easy to adapt for resource-constrained IoT environments. In the existing trust-based security for RPL routing attacks, nodes’ mobility is not considered or limited to only the sender nodes. Similarly, these trust-based protocols are not evaluated for mobile IoT environments, particularly regarding RPL attacks. Hence, a trust and mobility-based secure routing protocol is proposed, termed as SMTrust, by critically analysing the trust metrics involving the mobility-based metrics in IoT. SMTrust intends to provide security against RPL Rank and Blackhole attacks. The proposed protocol is evaluated in three different scenarios, including static and mobile nodes in an IoT network. SMTrust is compared with the default RPL objective function, Minimum Rank with Hysteresis Objective Function (MRHOF), SecTrust, DCTM, and MRTS. The evaluation results indicate that the proposed protocol outperforms with respect to packet loss rate, throughput, and topology stability. Moreover, SMTrust is validated using routing protocol requirements analysis to ensure that it fulfils the consistency, optimality, and loop-freeness.

## 1. Introduction

Internet of Things (IoT) is a network of smart objects connected with each other and the Internet. In IoT, smart devices exchange information and process data. IoT devices and applications are increasing exponentially [1]. The rise of IoT is capturing an important place in human life [2,3]. Thus, in recent years, smart devices and applications have seen incredible progress [4]. However, security and privacy concerns are a major hurdle in the widespread adoption of IoT. The substantial exchange of data is crucial in IoT networks and it is prone to breaching attacks. In sensitive applications, for example, healthcare, the security of networks, and data becomes a critical concern for end-users as well as the service providers. Due to inadequate security solutions in IoT, a number of disruptive attacks have been reported in recent years [5,6,7,8]. In addition, with the expansion and immense growth of IoT, the security attacks and their effects will increase in future [9,10,11]. Hence, IoT requires the fulfilment of the security triad, confidentiality, integrity, and availability (CIA) for its successful adoption.

**Figure 1:**
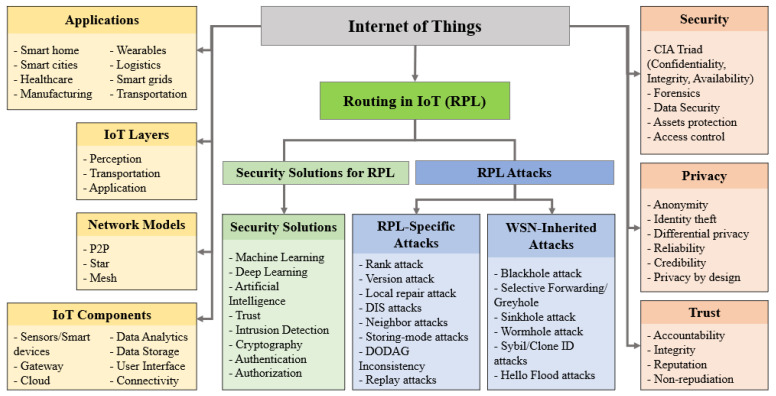
An Overview of IoT Applications, Layers, Network Models, Components, Security, and RPL Attacks.

For routing in 6LoWPAN and IoT, a variety of solutions are proposed. The IPv6 Routing Protocol for Low Power and Lossy Networks (RPL) is a standard introduced by the Internet Engineering Task Force (IETF) [12] to handle the routing requirements of smart devices and networks effectively. Like other protocols, however, RPL is prone to a considerable number of routing attacks [13] involving RPL-specific attacks, based on RPL features, and WSN-inherited attacks, that is, attacks derived from wireless sensor networks (WSNs) [14] because both IoT and WSNs domains are related to each other [15]. In IoT networks, it is challenging to detect and defend routing attacks because of devices’ nature and IoT systems’ unique requirements [16]. Figure 1 illustrates an overview of IoT applications, layers, network models, components, security, privacy, and trust, including the RPL attacks and existing security techniques.

This research work intends to focus specifically on the security of the RPL routing protocol. The routing attacks considered in this research include the Rank attack and the Blackhole attack in RPL. These two attacks have a high impact on routing process, among others. Fake rank information is advertised by a malicious node in a Rank attack. As a result, the routing information is forwarded to the sink node through malicious nodes by selecting it as a preferred parent. Similarly, the occurrence of Blackhole attack in routing causes dropping of packets and data loss. The widespread adoption of RPL protocol in smart healthcare, smart homes, smart cities, and the smart world [17] appeals that it is imperative to investigate these security attacks [18].

In the existing literature, numerous methods for solving the security of IoT routing are suggested, such as machine learning [19], intrusion detection systems (IDS) [20], trust [21,22], and other mitigation approaches [23,24]. The conventional security practices are inadequate to address the specific security needs of IoT. Because of its simple deployment and incorporation into the IoT network, the trust-based approach is a feasible alternative to provide RPL security. Furthermore, node mobility is not considered in current trust-based methods [25,26] or is inadequate for mobile sink nodes [27], especially for defence against Blackhole and Rank attacks in RPL. Hence, the proposed model scrutinizes the mobility metrics for trust computation. In addition, the proposed solution is investigated and evaluated for a dynamic IoT environment, considering mobile sender nodes along with mobile sink nodes.

This research aims to develop a trust-based routing protocol for IoT applications and improve security in RPL at the IoT network layer against Rank and Blackhole attacks. In this research work, we have selected the trust metrics through critical analysis and investigated their suitability for improving security in RPL routing protocol in mobile IoT environment [14]. SMTrust is integrated into RPL to provide secure routing with nodes mobility and enhanced network performance. The trust-based approach is applied to secure IoT networks, specifically routing, by detecting and isolating malicious nodes. SMTrust routing algorithms are embedded into RPL, and the protocol is evaluated for network performance, in terms of topology stability, packet loss rate, throughput, and power consumption. Overall, the performance of SMTrust is significantly better as compared to standard RPL objective function (OF), which is Minimum Rank with Hysteresis Objective Function (MRHOF) [28], and existing trust-based approaches, such as SecTrust [25], Dynamic and Comprehensive Trust Model (DCTM) [27], and Metric-based RPL Trustworthiness Scheme (MRTS) [26]. Following are the objectives of this research work:

1. To analyse and quantify the trust metrics and present an integration of SMTrust, proposed in [29], with RPL.
2. To provide algorithmic details for the integration of SMTrust into RPL.
3. To evaluate the network performance metrics via simulation.
4. To validate the simulation results and proposed protocol through mathematical analysis in accordance with the requirements of a routing protocol.

The summary of the main contributions of this research work includes the analysis and quantification of trust-based metrics to develop a trust and mobility-based routing protocol for secure routing in IoT. It is demonstrated that the selected trust metrics promote secure routing and improve the packet success rate, throughput, and routing topology stability. The proposed trust and mobility-based protocol will help in enhancing the defence against routing attacks in IoT. In general, this research will extend support to research community of IoT networks and routing security.

This research article is organized as follows. Section 2 provides the background knowledge of IoT networks, security, routing, and the significance of trust-based solutions. Section 3 refers and describes the related work. Section 4 presents the design and implementation of SMTrust, along with its integration with RPL. Section 5 elaborates the simulation results and analysis. Section 6 provides the validation and mathematical analysis of the proposed model, and Section 7 presents the discussion. Finally, the last section concludes the paper with some future directions of the research work.

## 2. Background

### 2.1. Routing Security in IoT

IoT layers are susceptible to various security attacks [30], including node capturing, denial of service [31], fake node or Sybil attack [32], replay attack [33], side-channel attack [34], and routing threats in data forwarding process [35]. The network layer is mainly prone to several attacks, including the routing attacks that are more disruptive to the IoT networks. Mostly in IoT applications, routing is performed using RPL protocol, which is vulnerable to WSN-inherited attacks and RPL-specific attacks. IoT is gaining prominence in multiple application areas, where security, especially networks and routing security, is one of the key issues. Furthermore, RPL is designed specifically for IoT networks for the fulfilment of routing requirements with efficient consumption of resources.

RPL is a standard introduced by IETF ROLL working group, initially specified in RFC 6550 in 2012. There have been a few updates after RFC 6550. A recent update that leverages RFC 6550 for its routing operations is specified in RFC 9010 in April 2021. Additionally, there have been several RFCs released by IETF to expand the original RFC 6550. Some advanced and related documents include the routing metrics (RFC 6551), OF0 (RFC 6552), MRHOF (RFC 6719), the optimization of parent node selection [36], and security threat analysis for RPL (RFC 7416). In addition to the above, a specification on the design guidelines for routing metrics composition by IETF was introduced. Moreover, several active Internet-drafts for RPL observations and extensions, provided at https://datatracker.ietf.org/doc/active/#roll (accessed on 22 May 2022), are intended for discussions and highlighting issues to the specified standard for up-to-date research work and possible improvements in the original RPL standard. Since its standardization, several studies have pointed RPL’s limitations related to its efficiency, applicability, and security. Researchers have proposed various extensions for the enhancement of the standard RPL specifications from implementation and operation point of view. This research work focuses only on the security attacks, such as Blackhole and Rank attacks, which have a high impact in RPL. The domain and focus of currently active IETF drafts vary from this research. Similarly, related to the security aspect, there has not been any implementation of RPL security features in any of the operating systems or platforms [37].

### 2.2. Attacks in RPL

RPL is prone to several attacks associated with its features inherited from WSNs, as explained in [14]. Since IoT is an enhanced type of WSN, some attacks are thus inherited from the layout and execution of routing in WSNs. In this research, only Rank and Blackhole attacks are considered, as they are severely disruptive and have a high impact on routing topology.

#### 2.2.1. Rank Attack

**Figure 2:**
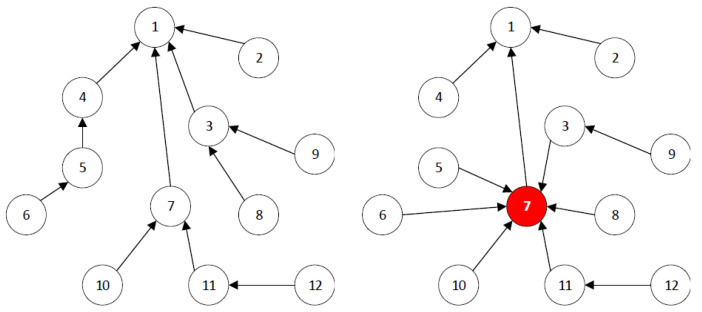
Normal Topology (**left** side) and Decreased Rank Attack (**right** side) [38].

The fundamental property of RPL is rank. It supports efficient and appropriate routing operations. Rank keeps track of control overhead, optimizes network topologies, and eliminates loop creation. An attack on the property of Rank has a major effect on RPL’s overall function and routing topology. There is no function in RPL to control the node’s behaviour, thus allowing chances for the occurrence of Rank attacks. The node picks a preferred parent depending on the objective function, and its rank value is computed as broadcasted by DIO messages in RPL routing. In the DODAG, the rank increases in the downward direction, that is, from the root towards the leaf node. A rank attack takes place when an attacker manipulates the rank values. Rank attacks can be divided into three categories. Worst-Parent, Increased Rank, and Decreased Rank attack. This research work focuses on defence against Decreased Rank attack. In a Decreased Rank Attack, the adversary is chosen as a preferred parent after lower rank is advertised by malicious node(s) to other nodes, as indicated in Figure 2. The effect of a Decreased Rank attack triggers substantial disturbance to network traffic and DODAG, which may result in increased energy consumption [38].

Furthermore, A malicious node broadcasting a lower rank (decreased Rank attack), causes some DODAG’s portion to link to the root through the attacking node and also enables it to eavesdrop on that network traffic-forwarding segment. RPL can be targeted in a number of ways by an internal adversary. However, some of these attacks have a slight effect on the network, while others have a substantial impact. An attack that leads to the reconstruction of DAG, the depletion of nodes’ resources, or eavesdrops on network traffic has a significant impact. Modifying the DODAG node rank value is a type of internal adversary to fulfil such malicious targets. In this way, the adversary is able to advertise the fake rank value, thus causing a Rank attack in the network. It severely affects the network topology. Attacks on the topology can isolate a node or a subset of nodes in the RPL network, blocking them from communicating with other nodes. The effects of the Rank attack are packet delay, reduced packet delivery ratio, and generation of un-optimized paths and loops. Practically, the effects of Rank attack, particularly the delay of data delivery, in sensitive IoT applications, such as smart hospitals, healthcare monitoring, and smart vehicular infrastructure, is very critical and can lead to devastating consequences.

#### 2.2.2. Blackhole Attack

**Figure 3:**
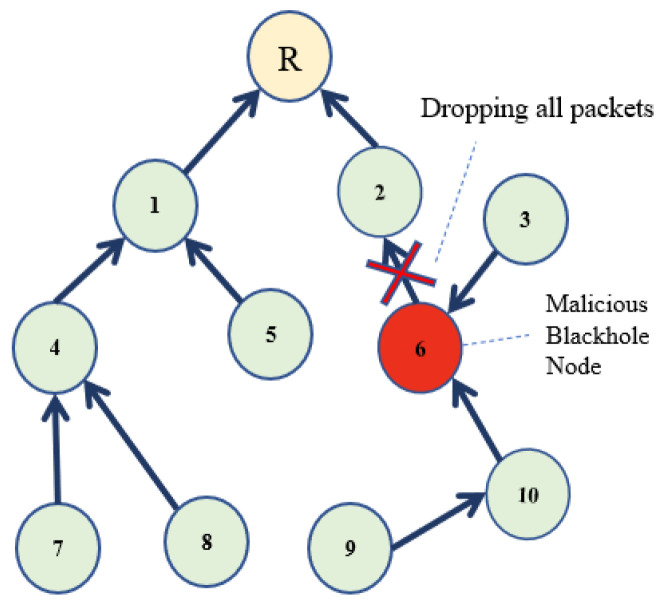
A Blackhole Attack Scenario in RPL [14].

Blackhole attack is among the severe and high-impact RPL attacks [39]. In a Blackhole attack, attacker node(s) drop all the packets, rather than forward to the destination, thus triggering a DoS attack in the network, as demonstrated in Figure 3. As an effect of the Blackhole attack, end-to-end (E2E) delay is increased, and packet delivery ratio (PDR) is reduced. If the packets are manipulated by the malicious node before forwarding, that variant of the Blackhole attack becomes even more devastating. It results in a misleading route advertisement in addition to a reduced PDR. The description and investigation of other RPL attacks are beyond the scope of this paper. Table 1 summarizes a description of the attacks under consideration.

## 3. Related Work

IoT devices in critical scenarios may generate a huge amount of sensitive data that requires protection from malicious access and tampering. IoT security is an evolving subject in research because of the new threats and apprehension of large-scale disruptions due to smart devices’ increased interconnectivity. Keeping in view the energy limitation and security aspects of the IoT devices, authors in [40] emphasized the energy-efficient fog computing solutions for massive IoT applications in 6G, and discussed the related challenges and opportunities. Similarly, to analyse the security enhancement, ref. [41] presented the derivation of optimal phase duration for artificial noise transmission based on security analysis.

Numerous security techniques and solutions [42,43,44,45] have been proposed for IoT security. The methods for addressing RPL attacks can be generally categorized as mitigation mechanisms and IDSs. The mitigation strategies rely on different approaches consisting of the methods based on identification, acknowledgment [46], trust, location, statistics/mathematics, and specifications. In addition, to maintain security, there are trust-based recommendation mechanisms presented [47].

Similarly, other mitigation methods for IoT routing attacks for RPL typically rely on either combining procedures to RPL or the modifications in current RPL, for instance, revising Objective Function (OF) [48]. Ref. [49] proposed a new OF by merging energy consumption and ETX along with the fuzzy logic method. Although the method demonstrates increased RPL efficiency in terms of packet delivery ratio, network lifespan, latency, convergence time, overhead, and energy usage. However, their work does not focus on addressing routing attacks.

### 3.1. Machine-Learning-Based Security Solutions

Machine Learning plays an important role and has been deeply studied by researchers for providing security solutions in different domains, including IoT [50]. Intrusion detection systems and machine learning approaches are effective from a security perspective, but their efficiency largely depends on provided datasets. IDS-based techniques for detecting routing attacks have certain flaws and limitations. For greater accuracy in attack detection, IDS-based approaches are changing from conventional machine learning to deep learning, particularly in sensor networks. However, none of the existing anomaly detection methods provide a mechanism to secure the overall system via attacks prevention [51]. Furthermore, achieving an effective true-positive-rate (TPR) and false-positive-rate (FPR) in real-time with minimal resource consumption is another intrusion detection challenge in the WSN context, for example, 6LoWPAN [52].

A trust-based security solution is feasible due to its lightweight characteristic, low computational cost suitable for IoT devices, and effectiveness for defending WSN-inherited (Blackhole attack) and RPL-specific (Rank attack) attacks [38].

### 3.2. Trust-Based Security Solutions

Trust-based approaches and trust modelling are feasible methods to countermeasure the security attacks in IoT networks. To make the system more reliable and trustworthy for users, trust modelling can be applied to the development of functional measures. Trust embedded in IoT networks and routing is significant for stability and security. This can enable network maintenance, particularly with a rise in the number of connected devices. In IoT networks and routing, trust-based approaches have not been thoroughly explored and pose a vital field of research, especially from the perspective of security. Additionally, the importance of trust models for IoT network and routing security, as explained by [14], indicates their implications.

The measurements of trust can be based on device or application and depend on the purpose and idea of the system [53]. Ref. [54] mentions that the trust metrics are based on either social or quality-of-service (QoS) properties. Correspondingly, nodes’ mobility is an important aspect to be considered while selecting and analysing the trust metrics in IoT. In a mobile IoT environment, contextual information involves device location as one of the metric categories. For instance, devices with known locations are more trustworthy than others. Similarly, the mobility-based metrics are helpful in trust computations. This concept is used more commonly in the case of VANETs. For example, in the trust model proposed by [55], time, location, and distance are considered important trust metrics.

A trust-based mechanism for RPL security, SecTrust [25], is proposed. It specifies its own OF to establish trust in RPL. The network efficiency and packet loss rate are evaluated considering the Rank and Sybil attacks. Similarly, a SecTrust revision is proposed [56] to evaluate Blackhole attacks. In addition, refs. [27,57] proposed a trust model for IoT, using QoS, quality-of-P2P-communication (QPC), and contextual knowledge for trust evaluation. The model is evaluated for Rank, Blackhole, and Sybil attacks in RPL.

A trust-aware and cooperative routing protocol, MRTS, is proposed by [26]. Trust calculation in MRTS is carried out by adding ETX metric. Their proposed approach is effective for packet delivery ratio (PDR), energy consumption, throughput, and node rank change. However, MRTS uses an IDS approach for attack detection and isolation, which is computationally expensive and requires a hardware security chip embedded in each node [58]. These approaches have a common research gap regarding the lack of consideration for mobility of nodes. Only [27] considers the mobility of nodes, but their evaluation is limited to sender nodes’ mobility. Ref. [59] presents a security protocol for trust management in IoT networks, but its focus is not on the routing attacks and nodes’ mobility.

The methods proposed in [46] insert a dummy packet in the network to mitigate the packet dropping attacks. The insertion of dummy packets creates high overhead, and this approach does not consider RPL-specific attacks. Thus, the existing trust models are not adequate from the perspective of mobile IoT environments. For attack detection and defence against malicious nodes, the trust-based approach is easily adaptable to IoT scenarios. It is also scalable at any node density level and can cope well with network size growth or shrinkage [60]. Moreover, it is important to select adequate trust metrics for an efficient security approach for RPL-based IoT.

In the existing literature, the trust models proposed for routing security in IoT lack some features, such as consideration of IoT node mobility, heterogeneity in IoT environments, adaptability to IoT networks and routing, and the consideration of RPL-specific attacks. Furthermore, trust dynamics and network performance are not taken into account in some of the presented solutions. However, some of them focus solely on network performance and routing behaviour, neglecting to address routing attacks and security concerns. Moreover, critical security attacks, particularly routing attacks in RPL, are not evaluated in some trust-based network security solutions. Table 2 shows the highlights of the most related works for this study, including the shortcomings of the existing trust-based solutions. Hence, it can be figured out that the existing trust-based approaches for RPL security are not adequate from the perspective of the mobile IoT environment.

**Table 2:** Related Work for Trust-Based Methods for Routing Security.

Ref.	Domain	Solution Technique	Evaluation Model	Mobility	Trust Metrics	Attacks Considered	Research Gaps/Improvements
[26]	RPL attacks/IDS-based	ETX for calculating trust for routing topology.	Simulation—Contiki2.7/Cooja	🗴	Energy; Honesty; Selfishness; ETX; Recommended Trust	Rank; Blackhole	Does not consider nodes’ mobility; Uses IDS-based attacks detection, and hardware security chip with each node.
[27]	RPL attacks	To combat RPL attacks, it includes a multidimensional and dynamic, trust model.	Simulation—Contiki/Cooja 3.0	🗸	QPC; QoS; Contextual information	Rank; Sybil; Blackhole;	Does not consider the mobility of sink nodes and its effect on the network.
[59]	IoT Protocols Security/IoT nodes/IoT networks	A protocol based on fuzzy logic, and a secure messaging system is developed.	Simulation—Contiki/Cooja	🗴	Direct trust; Indirect trust; Routing score	Bad service providers; Contradictory behaviour; On-Off attacks	Does not consider the nodes’ mobility and routing attacks.
[25]	RPL attacks	fuzzy logic-based approach.	Simulation and Testbed experimentation—Contiki/Cooja 3.0	🗴	Historical observation; Feedback; Successful and unsuccessful node transaction	Rank; Sybil	Does not consider nodes’ mobility; packet loss rate is significant; E2E delays, energy consumption, and colluding attacks are not evaluated; Does not consider the uncertainty of recommendations.
[46]	Wireless Networks/Routing Attacks	Dummy packets insertion.	Simulation—NS-2 for MANET, Cooja/Contiki 2.7 for IoT	🗸	Uncertainty; Contextual factors; Direct and Indirect trust; Route Trust	Packet dropping attacks	Inserts a dummy packet in networks creating high overhead; RPL-specific attacks are not considered.
[61]	Medical IoT mobility	A routing method for security in an energy-efficient sensor network	None	🗸	Energy consumption; Node capacity	Greyhole	Focus is on the energy consumption during data collection process and not on security and routing attacks.

As compared to the traditional security solutions, the use of trust in IoT networks can reduce the uncertainty factor for nodes interconnection. The trust models proposed for IoT lack certain characteristics, including heterogeneity, dynamicity, and resource-constrained nature of IoT [62]. Some trust models involve computationally expensive IDS-based approaches for attacks detection, which require a hardware security chip embedded in the device [26]. Unlike existing research, a comprehensive trust and mobility-based routing protocol is proposed that considers the high-impact attacks, such as Rank and Blackhole attacks, node mobility in an IoT environment, and the selection of appropriate trust as well as mobility-based metrics for an effective security solution for RPL-based IoT networks.

In proposed SMTrust secure routing protocol, the trust is computed considering the appropriate trust metrics, including the mobility-based metrics for nodes. This will facilitate the trust relationship between the nodes based on their mobility scenarios. Moreover, our proposed protocol is evaluated in static as well mobile scenarios for performance, considering the high impact attacks, such as Blackhole (WSN-inherited attack) and Rank (RPL-specific attack) attacks. The proposed solution, SMTrust, is different from the related methods basically for considering mobility metrics for trust computation, evaluation, and enhanced performance for static and mobile IoT environments.

## 4. SMTrust Design and Analysis

Mostly, the IoT devices are resource-constrained in nature. A trust-based security approach is viable because it is considered to be lightweight and effective particularly for defence against RPL-specific and WSN-inherited attacks [38]. In networks and IoT environment, trust metrics help in monitoring and control the interaction between nodes. Thus, for trust management in IoT, the selection of trust metrics is crucial for accurate evaluation and calculation of trust [55] The trust measurements may vary with the type of applications, devices, and purpose [53]. In RPL routing topology, the appropriate parameters, refer to as trust metrics, play a vital role in a trustworthy route establishment, thus supporting the packet forwarding to the reliable nodes and protecting the legitimate nodes from the anomalous behaviour of the malicious nodes. In addition, in a vulnerable network, the trustworthy interaction among nodes [63] enhances the overall performance and packets delivery in the network, while combating against the routing attacks.

**Figure 4:**
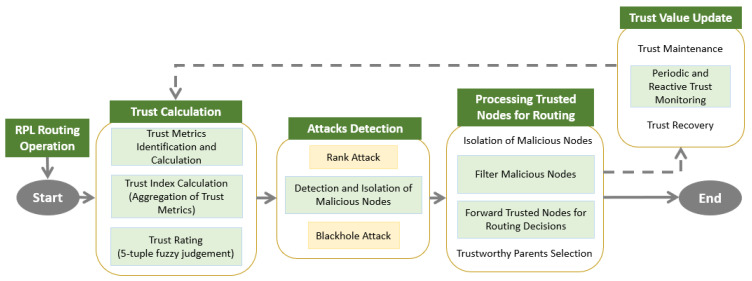
Flow Diagram of SMTrust.

This section presents the design specifications of the proposed trust and mobility-based secure routing protocol, SMTrust. A node’s reliability is determined by the direct and indirect trust values. The proposed method’s distinctiveness is that suitable mobility metrics are considered for trust evaluation to adapt to a mobile IoT environment. The diagrammatic model and workflow of SMTrust is based on the concepts detailed in [29]. The main components include trust metrics computation, trust index calculation, trust rating, trust value update, attacks detection, malicious nodes isolation, and trust decay and maintenance. Figure 4 depicts the diagrammatic flow of SMTrust.

### 4.1. Trust Metrics Identification and Trust Computation

Trust parameters are vital for the evaluation of a trust-based approach. The trust metrics employed by SMTrust are scrutinized in detail [14]. The important and applicable trust metrics in IoT involve historical observations, energy, direct trust, and recommendations, that is, recommended or indirect trust from neighbouring nodes. SMTrust incorporates mobility of nodes by considering mobility-based metrics in trust computation, including node relocation and link stability.

### 4.2. Quantification of Trust Metrics

Through the critical analysis of the trust metrics for security in IoT routing, six metrics have been selected for the development of the proposed trust-based solution. Direct trust is determined by the success rate (*TM_SR_*) of the node. *SR* is the ratio of the number of packets forwarded (*P_F_*) to the number of packets received (*P_R_*). This is referred to as direct trust or reliability of the node and the node’s suitability of effective communication in the network. Let *N_A_* be the trustor node (the evaluator) and *N_B_* be the trustee node (for which the trust is being evaluated), *P_R_* be the number of packets received by *N_B_*, *P_D_* be the number of packets dropped, then *TM_SR_* determines the overall success rate of the node calculated by Equation (1).

*TM_SR_ = P_F_/P_R_   , where P_F_ = P_R_ − P_D_* (1)

Similarly, historical observations (*H*_0_) are based on the recent trust value calculated for *N_B_* by node *N_A_*, if this is the first-time trust index calculation for node *N_B_* by node *N_A_*, then the value of *H_0_* will be set to 1. This supports the assumption that all nodes join the network as trusted nodes. It also satisfies that the node is not suddenly assigned a bad or good trust reputation in the network, rather it develops gradually.

*TM(H*_0_*) = Recently observed trust value of N_B_* (2)

The energy level (*TM_EL_*) of a node is revealed by the ratio of the remaining energy (*E_R_*) of the node to the maximum energy (*E_M_*) or battery capacity of the node, calculated by Equation (3). The maximum value of *TM_EL_* is 1 for a node with full battery.

*TM_EL_ = E_R_/E_M_* (3)

The location of the node determines that its position is not ambiguous and is clearer. Moreover, the distance between the trustor node (*N_A_*) and trustee node (*N_B_*) shall play a part for the closeness of the two nodes. The closer the nodes, the better is the link stability for successful packets transmission. In the scenario of wireless networking, it is vital to consider the quality and stability of the link, preferably the wireless signal strength for the trustworthy parent selection. Received Signal Strength Indicator (RSSI) quantifies the wireless signal strength. Adding this factor shall enhance the calculation of the trust index. Moreover, the RSSI value indicates the signal strength; a higher RSSI means that the signal is stronger and the node position is closer. Hence the trust metric for location and link stability (*TM_LLS_*) is determined by Equation (4).

*TM_LLS_ = RSSI value* (4)

In trust computation, mobility is defined as the number of relocations the node has gone through from a previously noted position. The less the number of relocations, the more reliable is the node. Suppose that the previously noted location of trustee node *N_B_* is *L_P_* and the current location of *N_B_* is *L_N_*, then mobility trust metric is calculated by Equation (5).

*TM_Mobility_ = Distance (L_N_, L_P_)* (5)

Recommended trust (*TM_RT_*) depends on the neighbouring nodes’ feedback or observation about trust of node *N_B_* being evaluated by node *N_A_*. This is calculated as the average of all the trust recommendation values for *N_B_* that node *N_A_* received (in DIO messages) from its *n* neighbours.

*TM_RT (NA, NB)_* = Σ _(1−*n*)_
*TrustIndex (N_n_, N_B_)/n* (6)

Recommended trust can serve the purpose for trust calculation is two-fold; one is that it can be used in the weighted calculation of trust index value of trustee node, that is, the node being evaluated. Secondly, it can be used to keep an additional record for the one-hop neighbours’ trust value.

### 4.3. Trust Index Calculation

Trust metrics are aggregated according to the assigned weights, and trust index is calculated. Current trust values are given more weightage (0.18) than historical observations (0.10). The success rate, energy, location, mobility, and recommended trust are given equivalent weightage of 0.18 because these are based on the current observations of the nodes. Whereas the historical observation is given less weightage of 0.10 because it is based on past behaviour but still is essential to take into consideration to develop the trust relationship gradually and not spontaneously. The aggregated trust values are ranging from 0 to 1. SMTrust uses the notion of a fuzzy threshold-based mechanism for the evaluation of trust ratings [64]. As evaluated by fuzzy judgment, the degree of trust index is five-tuple and is presented in Table 3 below. The tuple *t3* is used as the trust threshold.

SMTrust does not consider the nodes that lie in *t1* and *t2* tuple for routing decisions and secure communication. The nodes in tuple *t4* and *t5* are considered as reliable and trustworthy to be forwarded for routing decisions. However, the nodes included in *t3* are in the middle of *“No Trust”* and *“Full Trust”*. Hence, SMTrust tends to consider the nodes in *t3* when there is a shortage or no nodes available in the range of *t4* and *t5* tuple for communication, following the strict calculation of the trust index. This is also to overcome the non-availability of nodes for routing. So, the trust threshold is kept at 0.46, which is tuple *t3*. Moreover, the nodes with higher trust values are selected first for routing.

### 4.4. Rank and Trust Computation

Rank computation in SMTrust is adopted from the IETF RFC 6550 RPL specification [12] and as implemented in ContikiRPL default OF. Trust computation assumes that all nodes are trustworthy when joining the network. As ContikiRPL assumes that nodes overhear neighbour nodes and their packet transmission. The same assumption is made by SMTrust. Trust metrics are quantified in combination with the specification of rank for the selection of parent in routing. The OF specifies the routing decisions, including the preferred parent selection, and rank computation, according to the normal operations of RPL [65].

Once the topology is constructed, and the neighbour list is established, the trust values are computed according to the Equations (1) to (6) and the trust index is calculated accordingly. When DIO messages are received from neighbour nodes, the information communicated is used to update the routing table. The trust values of its neighbours are calculated using the SMTrust trust computation mechanism. A set of trusted potential parents is then selected for an optimal path to the root node. This process is followed by the neighbouring nodes for the construction of DODAG. Afterward, the DODAG maintenance relies on the trickle timer, which limits the frequent transmission of control messages. The trust value is updated periodically, using the trickle timer in RPL, and reactively, when there is a change in the node’s behaviour.

### 4.5. Attacks Detection

According to the definition and detection of a Rank attack and Blackhole attack, SMTrust adopts the concept of attack detection algorithms from [25,66]. This subsection explains the proposed attacks detection algorithms.

#### 4.5.1. Rank Attack Detection

In SMTrust Rank attack detection, when a node receives a DIO message from a neighbouring node, it checks whether the rank changes without DIO_seq. If the new DIO_seq is less than or equal to the current DIO_seq, it means that it is a suspicious or fake DIO [66]. Therefore, a Rank attack is identified. Moreover, checking the inconsistency of the rank of the potential parent against the neighbouring nodes indicates a fake DIO and Rank value [25,66]. Algorithm 1 demonstrates the detection of Rank attack in SMTrust.

#### 4.5.2. Blackhole Attack Detection

Blackhole attack is a kind of packet dropping attacks. SMTrust accomplishes this by overhearing and monitoring the neighbouring nodes as to whether the packets are being forwarded successfully or dropped by the trustee node. A Blackhole attack is detected by the trust index of the preferred parent node. Moreover, an additional check is the success rate in order to know that the packets are dropped by the node and to confirm a packet dropping attack. Algorithm 2 illustrates the detection of Blackhole attack in SMTrust.
**Algorithm 1.** Rank Attack Detection.**Input:** Set of potential parents $\left[p_{1},p_{2},\;p_{3},\;\ldots ,\;p_{n}\right]$ for node $N_{i}$, preferred parent, NeighborList
**Output:** preferred parent**BEGIN** for all p=1 to n belongs to the potential parents list do://check the validity of rank against NeighborList.   if (PotentialParents(p).DIO_seq=NeighborList.DIO_seq )    if (PotentialParents(p).Rank=NeighborList.Rank)     return preferredParent     //preferredParent selected is good    else     //preferredParent is not trustworthy. Rank attack detection.     Add PotentialParents(p)in suspiciousList[  ]     //isolation     preferredParent=NULL     **Go to** parent selection procedure   end if   return preferredParent**END**
**Algorithm 2.** Blackhole Attack Detection.**Input:** Set of potential parents $\left[p_{1},p_{2},\;p_{3},\;\ldots ,\;p_{n}\right]$ for node $N_{i}$, preferred parent, NeighborList
**Output:** preferred parent**BEGIN** for all p=1 to n belongs to the potential parents list do://check the validity of rank against NeighborList. $if\;(PotentialParents\left(p\right).T_{SR}$     $\geq SuccessThreshold)\&\&(PotentialParents\left(p\right).TrustIndex\left(N_{i},p\right)$     ≥TrustThreshold)  return preferredParent  //preferredParent selected is good else  //preferredParent is not trustworthy. Blackhole attack detection.    Add PotentialParents(p)in suspiciousList[  ]  //isolationpreferredParent=NULL  **Go to** parent selection procedure end if return preferredParent**END**

The proposed attacks detection algorithms slightly increase the complexity of the default objective function in RPL, because of the additional loop and check. In the Rank attack detection algorithm, the loop will be executed for all the potential *n* parents to check the validity of ran. So the complexity will be *O(n)* for the worst case, and if there is only one parent, the complexity will be *O(1)*, which is the best case. Similarly, in the Blackhole attack detection algorithm the loop will execute for all the parents from *p =* 1 to *n*, to check the success threshold and the trust index of the potential parents. The complexity of the Blackhole attack detection algorithm will also be *O(1)* in the best case if there is only one potential parent, and *O(n)* in the worst case of *n* potential parents.

The attack detection algorithms will check the validity of the potential parents unless it finds a trustworthy preferred parent. Once a preferred parent is selected, it will be checked for malicious behaviour through Rank and Blackhole attacks detection. If the node fulfils the specified conditions, it will be deduced that the preferred parent selected is good. Otherwise, the potential parent node will be added to the suspicious list, and the parent selection procedure will be called for the selection of another preferred parent from the potential parents list.

## 5. Simulation Study

**Figure 5:**
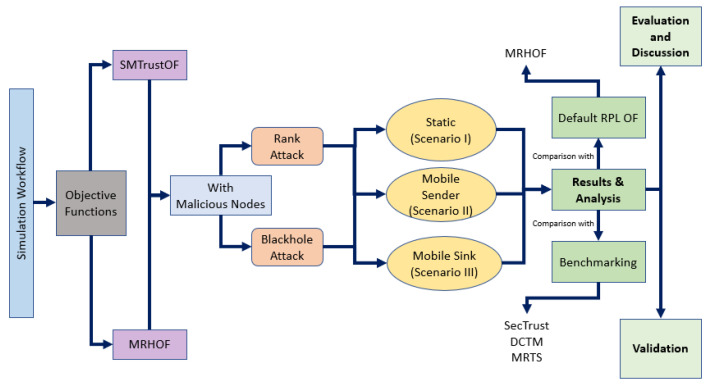
Simulation workflow.

The proposed trust and mobility-based routing protocol is tested via simulation study by embedding in RPL routing protocol. This is carried out by using ContikiOS/Cooja simulator with InstantContiki2.7. SMTrust is tested under three scenarios: Scenario I of static nodes, Scenario II of mobile sender nodes, and Scenario III of the mobile sink and sender nodes. The simulation performance of SMTrust and default ContikiRPL, along with existing systems, under routing attacks is determined and analysed for parameters, including node rank changes, packet loss rate, throughput, and power consumption. Figure 5 shows the simulation workflow of our experiments.

Contiki [67] is an open source, wireless sensor network operating system consisting of a kernel, program loader, and several libraries and processes. Contiki OS is the most commonly used platform [68], which contains distinct modules for different tasks, and all tasks are divided logically into directories. This research work focuses on the routing module, that is ContikiRPL, in Contiki OS. Hence the coding is mainly carried out in the *rpl* directory, located at *…/contiki/core/net/rpl*, to implement and integrate the proposed trust-based secure routing system. So, the Contiki OS is chosen because it provides enough flexibility of code modification in the required directory and supporting mobility scenarios.

The implementation of ContikiRPL is according to the specification in RFC 6550 [12]. Two objective functions are implemented in ContikiRPL, Objective Function Zero (OF0) and the Minimum Rank with Hysteresis Objective Function (MRHOF). This research work defines its own objective function, named as SMTrustOF, and manipulates ContikiRPL according to the SMTrust model and process flow as depicted in [29]. In the proposed secure routing protocol, a comparison is made with MRHOF because OF0 is already proved to have lower performance in RPL implementation [69,70]. Using SMTrustOF, the routing decisions rely on the trust among nodes, thus detecting and isolating the malicious nodes in the network. All nodes calculate trust values for the neighbouring nodes to choose only the trustworthy nodes for routing. The trust values evolve over time according to the weightage assigned to each trust metric.

### 5.1. Experimental Setup

The topology is generated using 30 sky nodes, including three attacker nodes, twenty-six sender nodes, and one sink node, assuming that up to 10% of nodes can act as malicious nodes in a typical network environment. According to the existing literature, the assumption of 10% attacker nodes is feasible for the simulation experiments and SMTrust evaluation. For a typical IoT application today, for example, smart home or smart hospital, a network of 20 to 30 nodes is common [25,27]. However, this number could arguably increase in future, according to the predictions. Attacker nodes are positioned randomly among the normal nodes to demonstrate a real-use case scenario where attacker nodes can intrude on the internal smart environment network.

#### 5.1.1. Mobility

Stationary as well as mobile devices are used a great deal in IoT applications, for example, in the smart hospital context. These devices include mobile devices, wearable external devices, stationary, implantable, and supportive devices [71]. Therefore, the ratio of mobile to static nodes is kept at 1:3 (approximately) to depict the real-world scenario of a simple smart home or smart hospital application. In order to evaluate the SMTrustOF performance in a mobile IoT environment, a mobility plugin with Cooja is used. The BonnMotion-3.0.1 tool [72] was used to produce the mobility scenario for the nodes using the Random Waypoint mobility model. The output of the BonnMotion tool is converted using a built-in application WiseML to make it compatible with the Cooja supported mobility extension movement file.

#### 5.1.2. Parameters Setting

The reception ratio (RX) value is set to a variable range of 30–100% to illustrate nodes’ lossy nature. The Transmission ratio (TX) set to 100% for all nodes, depicting a loss-free transmission as the introduction of losses was only needed at the reception end but not at the radio transmission end. For all devices, the transmission range was set to 50 m and the interference range as 60 m. The interference and transmission ranges are in accordance with the coverage range of the available sensor radios. The simulation was carried out for 60 min. Each node periodically sends a 46-byte packet to the sink, including 30 bytes data and 16 bytes frame header, that is every 60 s (1 min) and after an initial start-up delay of 5 s. For the RPL network to converge, the initial start-up delay was set to 5000 milliseconds (5 s), which is appropriate for the RPL network according to the convergence time of 10 to 100 nodes [25,27].

RPL convergence time is required for all the nodes to join the network. It can be observed from the simulation platform when the DAG is completely constructed, and all the nodes have joined the network. In this way, the packets considered for evaluation are the packets after network convergence. While creating a new simulation in Contiki, the developer can set the mote start-up delay (ms) in advanced settings according to the requirements. ContikiRPL supports the storing mode of operation by default. Similarly, DIO doublings and DIO min is also kept by the default setting of ContikiRPL as 12 and 8, respectively.

In our experiments, we follow the default RPL operation and use the trickle timer for trust update and monitoring process. Trickle timer algorithm uses a periodic interval for sending out DIO messages to conserve nodes’ energy resources in the network. DIO min, in Trickle timer, is the minimum interval between any two DIOs. Similarly, DIO doublings determines when the maximum value of the interval is reached. SMTrust makes use of the trickle timers so as to bound the control packet overhead, that is, to reduce the number of control packets by eliminating redundant messages. The parameters’ setting in the Cooja simulator is adopted from our benchmarks [25,26,27], with the justification that this setting is feasible to evaluate a trust-based routing solution according to an IoT application depicting a real-world scenario.

### 5.2. Results and Analysis

**Figure 6:**
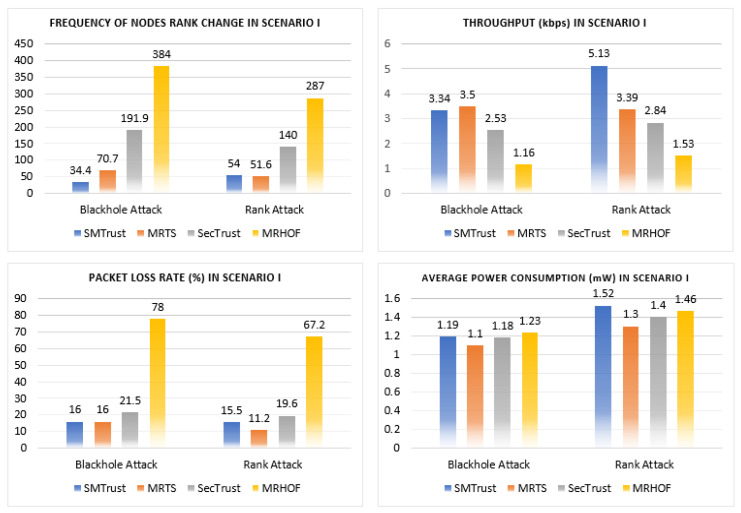
Results comparison of network performance under Blackhole and Rank attack in Scenario I.

**Figure 7:**
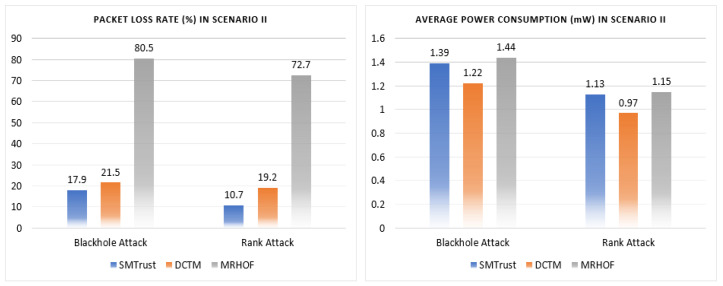
Results comparison of network performance under Blackhole and Rank attack in Scenario II.

**Figure 8:**
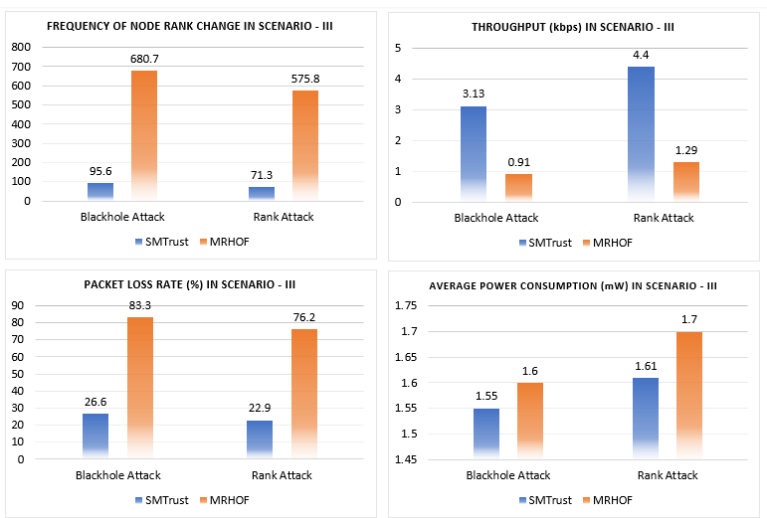
Results comparison of network performance under Blackhole and Rank attack in Scenario III.

In this section, the performance analysis of the proposed SMTrustOF-based protocol is presented in comparison with existing protocols. Multiple simulation runs under three different scenarios were used to verify the results. Figure 6, Figure 7 and Figure 8 present the average results for RPL topology, including 26 legitimate nodes, one sink node, and three attacker nodes. Scenario I indicates that there are only static nodes in the network. Scenario II means that there are mobile sender nodes in the network with a mobile to static ratio of 1:3, and a static sink node. Scenario III indicates that there are mobile sender nodes with a mobile to static ratio of 1:3, and the mobile sink node in the network. Four parameters are used for the performance evaluation of our proposed solution, that is, packet loss rate, frequency of node rank changes, throughput, and power consumption. A detailed analysis of results comparison is presented below.

#### 5.2.1. Network Performance Comparison in Scenario I

Scenario I comprises only static nodes in the network. In a Blackhole attack, the malicious nodes tend to drop the packets. Figure 6 shows the graphs for network performance analysis under Blackhole attack in Scenario I. The frequency of node rank changes determines the stability of the network. On average, the frequency of node rank changes is 54, using SMTrustOF under Rank attack in static nodes scenario. The average number of node rank change is quite justifiable in a RPL topology of 30 nodes with random positioning in the network, including the malicious nodes. Under Rank attack, SMTrust shows a stable topology as compared to SecTrust and MRHOF. However, compared to MRTS, SMTrust shows a negligible difference of 2.4 under a Rank attack in Scenario I. Similarly, SMTrust shows a more stable topology under Blackhole attack with an average frequency of node rank change as 34.4, as compared to SecTrust (191.9) and MRTS (70.7).

Similarly, the packet loss rate for SMTrust stays lower as compared to MRHOF. On average, SMTrust experienced a lower packet loss rate of 15.5% under Rank attack in Scenario I, indicating a promising defence against Rank attacks. In comparison with MRTS, under Blackhole attack, SMTrust shows an equivalent packet loss rate. However, there is a small difference for the results comparison of SMTrust with MRTS, which is 4.3% under Rank attack in Scenario I. This can be justified by the fact that MRTS uses IDS for attack detection, which is computationally intensive and needs a hardware security chip embedded in the device, whereas SMTrust does not need additional hardware for security because it uses a simplified trust computation mechanism.

The throughput measures in SMTrustOF-based RPL are quite satisfactory. The throughput of SMTrust is much higher as compared to MRHOF. Under a Rank attack, SMTrust shows a higher throughput as compared to MRTS (3.39 kbps), SecTrust (2.84 kbps), and MRHOF (1.53 kbps). The average value of throughput performance in SMTrust under Rank attack in static scenario is 5.13 kbps, indicating that the network is not segmented due to the Rank attack perpetrated by the malicious nodes in the network. It is also an indication that all the nodes communicated with the sink and the attacker nodes were successfully isolated from the network as untrusted nodes. For the average throughput value in Scenario I (Figure 6), as compared with MRTS, SecTrust, and MRHOF, SMTrust performs comparatively better than existing solutions, with 3.34 and 5.13 kbps, under Blackhole and Rank attacks, respectively.

For average power consumption, SMTrust shows similar results as compared to MRTS, SecTrust, and MRHOF under Blackhole and Rank attacks in Scenario I. The performance of SMTrust with respect to power consumption is because SMTrust considers the trust metrics computation and consumes a little more power in computations and DIO transmissions. However, once the malicious nodes are identified and isolated, the topology becomes stable and power consumption stabilizes as well.

#### 5.2.2. Network Performance Comparison in Scenario II

SMTrust shows even better results for Scenario II under Rank and Blackhole attacks due to the inclusion of mobility-based metrics in trust evaluation, as in Figure 7, thus indicating that the performance of SMTrustOF is much improved compared with MRHOF with mobile sender nodes in the network. Since the focus of DCTM is for mobile sender nodes in the network, the SMTrust performance is compared with DCTM under Rank and Blackhole attacks. For Scenario II, SMTrust is compared with DCTM and MRHOF under attacks. It can be observed from Figure 7 that the packet loss rate of SMTrust is 17.9% under Blackhole attack, which is much lower as compared to DCTM (21.5%). Similarly, under Rank attack, SMTrust shows an average packet loss rate of 10.7%, which is much lower than DCTM (19.2%). Similarly, the average power consumption of SMTrust shows only a negligible difference as compared to DCTM and MRHOF under attacks. Hence, overall SMTrust shows a better performance in Scenario II, comprising mobile sender nodes, under Blackhole and Rank attacks.

#### 5.2.3. Network Performance Comparison in Scenario III

Since there is no study in the existing literature which focuses on the mobility of sink nodes for evaluation of the security in RPL hence SMTrust performance is compared only with MRHOF. The results for Scenario III under Blackhole attacks, as depicted in Figure 8, indicate that SMTrust performs better than MRHOF for topology stability, packet loss rate, and throughput. The frequency of nodes rank change of SMTrust is quite stable with 95.6 and 71.3 under Blackhole and Rank attack, respectively, in Scenario III. The average packet loss rate of SMTrust for Scenario III under attacks is 26.6% and 22.9% shows significant improvement as compared to MRHOF (83.3% and 76.2%). This also shows that though the mobility of the sink node in Scenario III degrades the network, SMTrust still shows a lesser packet loss ratio. Similarly, the throughput of SMTrust (3.13 kbps and 4.4 kbps) is higher as compared to MRHOF (0.91 kbps and 1.29 kbps) under both attacks, indicating that the attack scenarios greatly affect the throughput for MRHOF, because of no defence mechanism. Moreover, in comparison with MRHOF, SMTrust shows a lesser average power consumption under Blackhole and Rank attacks in Scenario III, as compared to MRHOF.

#### 5.2.4. Comparison of Power Consumption

The power consumption depends on the nodes’ involvement in the network, for example the frequency of rank change, parent selection, and trust computation, so it cannot be consistent for all the nodes for three scenarios under attacks. This is reflected in the graphical analysis of average results for power consumption in Figure 6, Figure 7 and Figure 8 for Scenario I, II, and III, respectively. It can be observed that SMTrust shows a comparably acceptable power consumption value for all three scenarios, with an average difference of only 0.22 mW. This difference is due to the computations involved in trust metrics calculations and trust evaluation, including the control messages transmission. For this research, the improvement of power consumption is beyond the scope and will be considered in future work. The values for power consumption of nodes are taken from the sensor collect view in Cooja simulator. Some of the nodes consume more power than others because they are chosen as preferred parents more frequently.

In addition, the increased frequency of node rank changes, that is, topology instability, causes more power consumption, as in case of MRHOF. Similarly, according to the scenario for static nodes (Scenario I), SMTrust consumes more power as compared to other OFs, because of the computations involved; specifically, the mobility-based metrics for trust calculation is not applicable for static scenario. Whereas for Scenario II and Scenario III in which the nodes are mobile, the power consumption is less as compared to other OFs, because the mobility metrics involved for trust calculation play a part in parent selection according to neighbouring nodes’ mobile nature. IoT networks are lossy in nature, and for this research we have considered the packet loss rate under the attack scenario. It is worth noting that the packets can be lost due to link unreliability (lossy nature), collisions, or attack scenarios. Since isolating the packet loss due to collisions or link instability would be a much more complicated task to undertake, in our experiments we therefore reported the overall packet loss rate. This is justified because the parameters settings and simulation configurations remain the same for all the experiments.

From the average values, it can be analysed that SMTrust performs better than SecTrust and MRHOF in Scenarios I, II, and III under attacks. Overall, SMTrust offers more stable topology in all three scenarios under attacks in comparison with existing methods. Overall, SMTrust shows better performance for both Rank and Blackhole attacks. SMTrust performance is better for Scenario II in comparison with Scenario I and Scenario III, indicating the importance of mobility-based trust metrics computation for parent selection algorithm. Moreover, the effects of sink mobility degrade the network performance for SMTrust under Rank and Blackhole attacks compared to Scenario I and II results. However, SMTrust still outperforms in overall network performance parameters, including topology stability, packet loss rate, and throughput measurement of nodes, thus indicating significant improvement as compared to existing systems.

The major impacts of the considered RPL attacks include the increased packet loss and reduced throughput, which is evident from the results of the default objective function MRHOF, that is, the increase in packet loss rate, the reduction of average throughput, and the increase in the frequency of nodes rank change. It is to be noted that there is no attack defence mechanism in MRHOF, which is RPL’s default implementation. When SMTrust is integrated into RPL’s objective function, the results under attack scenarios show that the packet loss rate is reduced and throughput is increased as compared to MRHOF, thus justifying the detection of attacks by eliminating the malicious nodes from the potential parents list and adding to the suspicious list, leading to its isolation from the network when no traffic will pass through it. When the proposed protocol is implemented in the same attack environment, the frequency of node rank decreases, packet loss rate also decreases, and throughput is increased. This proves that the proposal is able to defend against the RPL attacks.

## 6. Validation of the Proposed Protocol

In this section, SMTrust is validated using the routing protocol requirements specified in [73,74]. RPL uses source routing or hop-by-hop forwarding to send packets to the root node. Additionally, RPL uses the Bellman–Ford algorithm to calculate path cost, because RPL is a distance vector-routing protocol [75]. The shortest path is computed by this algorithm from source to destination in a weighted directed graph. Thus, the SMTrust and trust index metric is analysed to fulfil the routing protocol requirements considering the theoretical framework by [74,76].

### 6.1. Routing Protocol Requirements Analysis

While selecting the metrics for a routing protocol, the routing requirements of consistency, optimality, and loop-freeness must be met [74]. The routing metrics requirements are also emphasized by the IETF Roll Working group [7]. Authors in [26] validate their proposed approach for secure routing protocol as per the study of [74], which says that for a routing protocol to operate properly, it should fulfil the abovementioned three requirements. Additionally, a packet forwarding scheme and a path calculation algorithm are the two main elements of a routing protocol. Similarly, isotonicity and monotonicity properties are required to be satisfied by a routing metric [73,74]. IETF ROLL [73] defines a composite metric as a routing metric that consists of basic and derived metrics imposing a purposeful mechanism or function.

#### 6.1.1. Network Model

Let SMTrust-based RPL network be defined by a Directed Acyclic Graph *G = (V, E)*, where set of vertices or nodes is denoted by *V* and set of edges or links is denoted by *E*. An edge *e = (i, j)* is linked with a trust index *T(i, j)*, where *e* ∈ *E* and *i, j* ∈ *V*, and *T(i, j)* is the trust index of the neighbouring node *j* as calculated by node *i*. In the SMTrust-based RPL network, the traffic is MP2P, where data is sent from the sender or source nodes to the sink or root node. Thus, traffic is upwards from *N_n_* nodes to *N_1_*, where *N_1_* is the sink node. Let *p* be the path from node *N_n_* to node *N_1_*, such that, *p<N_n_, N_1_> = (N_n_, N_n−1_, …, N_1_)*.

On the path *p<N_n_, N_1_>*, let *N_p_* be the next-hop node in the neighbour of *N_k_*_,_ on the path *p<N_n_, N_1_>*, where *k = n, n−1, …, 2*. Then *neighbor(N_p_, N_k_)* represents the 1-hop neighbor of node that *N_k_* selected as the preferred parent, storing it in routing table and using for traffic forwarding. The path calculation algorithm in SMTrust is the Bellman–Ford algorithm, as in the standard RPL protocol, represented by the function *path_calculation(G, f, sd, sn)*, which returns the path from source node *sd* to the sink node *sn*, *p<sd, sn>*. Two types of nodes are considered in our network model: trusted and suspicious nodes. The trusted nodes are the legitimate nodes with trust index *T(i, j) > = T(threshold)*, whereas the suspicious nodes have trust index *T(i, j) < T(threshold)*. A sub-path is defined as any portion of the path traversed between the source–destination nodes of any given pair.

#### 6.1.2. Consistency

As authors in [73,74] have mentioned, a routing protocol is consistent if each node consistently follows the packet forwarding decision. For example, if a node *N_n_* has selected a parent *N_p_* and the path chosen is *p<N_n_, N_1_>* where each node on this path should forward packets via sub-paths of this selected path. According to the specifications, it is observable that SMTrust is consistent. For source routing, packet headers are used to forward packets by the on-path nodes, thus the routing consistency is satisfied. Similarly, the consistency of hop-by-hop forwarding is satisfied as the tree or topology in SMTrust is constructed downwards from root node to leaf nodes. This indicates that each transitional node is selected as the preferred parent of any sender node on a specified path. Thus, if a neighbouring node *N_p_* is selected as a preferred parent by node *N_k_*, a sub-path towards sink node is inevitably selected because the sender node forward packets to the parent node first, that is, to node *N_p_*. The same method for packet forwarding is followed by each node on the sub-path. Thus, the SMTrust-based RPL network fulfils the routing requirement of consistency or network convergence.

#### 6.1.3. Optimality

A path that minimizes the rank value between a particular pair of source and destination nodes, along with the sub-paths, is defined as an optimal path [73]. A routing protocol is optimal if the traffic for each pair of nodes in the network is routed along the best path [76]. As SMTrust utilizes the *path_calculation(G, f, sd, sn)* function for the calculation of the path cost from sender to receiver *p<sd, sn>* and the best path is selected. The parent selection algorithms implied by SMTrust also indicate the selection of the best route towards the root node from a sender node, thus fulfilling the property of optimality.

#### 6.1.4. Loop-Freeness

In RPL, the DAG is constructed when each node selects its parent, calculates its rank accordingly, and broadcasts it to the neighbours via DIO messages. In this way, each node selects a node as a parent if it possesses the minimum rank value. Rank is a scalar representation of the node location within the DODAG. The rank value increases from the root towards the leaf node monotonically. Thus, the rank computation in RPL routing is sufficient to fulfil the condition of loop-freeness and consistency. Similarly, the calculation of valid and optimal paths and consistency ensures loop-freeness. In accordance with the analysis, SMTrust is consistent and hence loop-free, as it follows the standard RPL operation for rank computation. Additionally, when a path is selected, the rank is calculated by the source node based on the selected parent rank. So, when a DIO message is received from another node with a greater rank value, the receiver node discards that DIO message, thus avoiding the loops formation. However, there in the scenario where the routing loops occur because of a distributed Bellman–Ford algorithm [75], then the RPL protocol offers the global and local repair mechanisms accordingly.

### 6.2. Mathematical Model of SMTrust

There are certain properties that the routing metrics must hold to support the routing protocol requirements for RPL [73]. To guarantee that the routing protocol is appropriately operational, two properties are identified by [74] that need to be fulfilled through a routing metric, i.e., isotonicity and monotonicity. For a distributed Bellman–Ford algorithm-based proactive routing protocol, such as RPL, refs. [74,76] have elaborated the theorems for isotonicity and monotonicity properties to fulfil the consistency, optimality, and loop freeness. The monotonic property ensures the convergence and loop-freeness of the routing algorithm, whereas isotonicity, affecting the path weights, ensures the optimality. In addition, ref. [76] proves that isotonicity and monotonicity are adequate to ensure that the routing protocol is loop-free, optimal, and consistent. Ref. [73] also supports that a composite metric in a routing protocol must hold the properties of isotonicity and monotonicity. Following the work of [73,74,76], and as adopted by [26], these properties for SMTrust are explained below to analyse and validate the proposed model.

Let the routing metric algebra be defined by the quadruplet *(P, +, fn, ≤)* called the path weight structure. *P* is the set of paths, *fn* is the function mapping a path to weight, ≤ corresponds to costs order relationship that provides total order of weights, and *+* is the concatenation operator. For SMTrust, *fn* in path weight structure is path *p* trust value. Since SMTrust uses Bellman–Ford algorithm for path calculation, as in the standard RPL operation, and according to the Lemma and Theorems 7, 8, and 9 proved in Yang et al.’s study [74] and Sobrinho [76], we can demonstrate that SMTrust holds the (left) isotonic and (left) monotonic properties.

According to the specification RFC 6550, RPL is a path addressing and source routing the protocol, meaning that the sender partially or fully specifies the route of the packet in the network. Hence, the node is enabled to communicate all the potential routes to the receiver. Since SMTrust follows the standard RPL operation, different definitions can be used for path weight structure by capturing different path characteristics [74]. Hence, in path weight structure *(P, +, fn, ≤)* for SMTrust, *fn* is the function that is based on the maximum trust index value along the path *p*. The order relationship *≤* represents the minimizing trust relationship of paths. Meaning that for the trust index metric, ‘min’ is the metric operator and ‘max’ is the metric order relation. Thus, based on the proved theorems and lemma, provided that SMTrust relies on Bellman–Ford’s algorithm, it is obvious that SMTrust holds the (left) isotonic and (left) monotonic properties for the calculation of paths.

#### 6.2.1. Isotonicity

The isotonicity refers to preserving the order of the weights of two paths when prefixed by the same third path [73]. Mathematically, the path weight structure of SMTrust *(P, +, fn, ≤)* is (left) isotonic for paths *x, y, z* belongs to *P*, infers that if *fn(x) ≤ fn(y)* then *fn(z + x) ≤ fn(z + y)*.

#### 6.2.2. Monotonicity

The monotonicity refers to a metric that should ensure the increase in path weight if prefixed by another path [73]. Mathematically, the path weight structure of SMTrust *(P, +, fn, ≤)* is (left) monotonic for paths *x, y, z* belonging to *P*, infering that *fn(x) ≤ fn(z + x)* and *fn(y) ≤ fn(z + y)*.

Other than these, there are additional properties and rules, as described by [73], that the basic, derived, and composite metric must hold to ensure the routing protocol requirements. For the calculation of the trust index in SMTrust, the defined rules and properties are examined and followed accordingly. The explanation of the rest of the properties for SMTrust is beyond the scope of this paper.

## 7. Discussions and Findings

There has not yet been any trust-based system, in the existing literature, that has been examined for both static and mobile scenarios under attacks. Most of the existing trust-based systems for routing security are for a static environment, such as a smart home [25]. To the best of our knowledge, only one study, DCTM [27], has been tested for mobile sender nodes. However, the performance of trust-based secure routing protocol is not tested under a mobile sink node environment. The simulation performance for SMTrust under routing attacks is determined and analysed for various parameters, including node rank changes, power consumption, packet loss rate, and throughput. The results indicate that SMTrust outperforms MRHOF [28], SecTrust [25], DCTM [27], and MRTS [26] in terms of network performance under Rank and Blackhole attacks.

For power consumption, SMTrust shows a negligible increase as compared to existing systems. This is due to the difference in trust index computations, attack detection, and trusted parent selection. As compared to MRTS, SMTrust shows negligibly lesser throughput under Blackhole attack and higher packet loss rate under Rank attack. This is because MRTS uses an IDS approach for attack detection and isolation, as well as a hardware security chip embedded in each node. This is justified by the fact that SMTrust employs simplified equations to compute trust metrics and trust index, thus avoiding integration of IDS or hardware separately.

### 7.1. Computational Complexity

With reference to computational overhead and in comparison, with MRHOF, the overall complexity of SMTrustOF is increased. The computational complexity of MRHOF is O(1). For SMTrustOF, the objective function is modified, basically for trust-based parent selection and attacks detection. The complexity of the parent selection algorithm is O(1) except the trust calculation for neighbouring nodes required as input for parent selection, which is O(n). Similarly, the computational complexity for attack detection algorithms is O(1) in the best case and O(n) in the worst case. Therefore, it can be deduced that the overall complexity for the added procedures in SMTrustOF is O(n). For SMTrust, the computational complexity increases with the calculation of trust metrics and aggregation.

Similarly, in terms of memory overhead, additional memory is required for storing and aggregating the trust values. The procedures introduced for SMTrust include individual trust metrics calculation, as explained by the equations in Section 4.2, which are finally aggregated to calculate the trust index. This trust index is used in the parent selection algorithm for comparison of potential parents. The attack detection procedures are called on during the parent selection to validate the selected preferred parent. Undoubtedly, the proposed SMTrustOF increases the complexity of RPL in terms of computation, memory, and runtime. For space complexity, basically the trust metrics calculation, storage of historical observations, and recommended trust values from neighbours, are the additional memory consuming tasks involved in SMTrustOF. The data types of int, unsigned int, and float have been used to accomplish the above, and each occupies 4 bytes space size.

### 7.2. Limitations of Research

This research work focuses on the routing protocol security under Rank and Blackhole attacks in RPL-routing protocol. Though the results are promising, there are some limitations of the proposed secure routing protocol and experimentation that are worth mentioning for better understanding.

The calculation of trust recommendation requires recommended trust values from neighbouring nodes. A considerable number of IoT nodes in an area means that a node will have many neighbours, in which case the star topology is preferred. From a topology perspective, a star topology consists of the central connecting devices, which are the relay nodes responsible for disseminating the information to the appropriate host or destination. In the RPL specification, the information forwarding is based on the selection of a parent node, and each sender node must forward the packet to its preferred parent node, which is selected by rules specified in objective function. Since our research work is based on RPL-routing protocol specification and implementation, which follows the P2P network model, therefore for the evaluation of this research work, the topology is constructed according to the RPL standard specified by IETF in RFC 6550, keeping in view the current developments and implementations of the standard.

## 8. Conclusions

IoT is evolving in several application areas, resulting in billions of devices being interconnected. These devices are resource-constrained by nature and are unable to implement conventional security techniques of machine learning and IDS. RPL-routing protocol is adopted by most IoT applications and is prone to numerous attacks. To address these attacks, a trust-based approach is a viable option. This research proposes a trust- and mobility-based secure routing protocol, which relies on critically chosen trust metrics, including mobility metrics. The results indicate that the proposed solution outperforms the existing techniques with enhanced network performance for static and dynamic scenarios in topology stability, packet loss rate, and throughput. Moreover, it has been proven to fulfil the routing protocol requirements.

For future work, the proposed protocol shall be enhanced for power consumption. It will also be evaluated for colluding attacks. We plan to evaluate the results with an increased number of nodes in the network as well as with the testbed experiments considering additional performance parameters and improved experiments for real-world scenarios.

## Figures and Tables

**Table 1:** Description of Attacks Under Consideration for Proposed Secure Routing Protocol.

Attack	Description	CIA Impact	Effects on Network Performance
Rank Attack	The malicious node attracts traffic by advertising its fake rank value.	Confidentiality, Integrity	Severely affect the routing topology; Disruption in RPL’s DODAG and network traffic; Longer E2E delays; Curtail packet delivery ratio; Introduce routing loops and non-optimal paths.
Blackhole Attack	A malicious node may broadcast to have an optimal path, and once it receives traffic, it starts dropping packets.	Confidentiality, Integrity, Availability	Create a DoS inside the network; Dropping packets leads to data loss; Increase E2E delay and control overhead; Curtail packet delivery ratio; Increase route traffic.

**Table 3:** Five-Tuple Trust Rating.

Trust Index	Trust Rating	Trust Value Range
t1	No Trust	0.0–0.20
t2	Poor Trust	0.21–0.45
t3	Fair Trust	0.46–0.70
t4	Good Trust	0.71–0.90
t5	Full Trust	0.91–1.00

## Data Availability

Data will be provided on request.
